# On the Use of Digitalis in Consumption

**Published:** 1799-12

**Authors:** 

**Affiliations:** Birmingham


### *On the Use of Digitalis in Consumption: by* Dr. Bree, *of Birmingham.*

[Continued from p. 314—318 of our last Number.]

Case 9.—Mr. Ralph Ward, aged 30, tall and thin, with a long neck, was attacked in Sept. 1798, with pneumonia, which I treated in the usual manner. In the beginning of the winter, I found his pulse nearly as quick as when I prescribed for him in Sept. He had a short cough, and a tightness in his breast; he had been subject to a cuticular eruption, which I then endeavoured to promote; but though it appeared very plentifully all over his body, there was not the relief which I expected in his breast; his pulse was never slower then 96, generally 100, and he had a good deal of languor. In April 1799, he spat bloody mucus for a few days.

1799, May 21st. He has a short and frequent cough—shooting pains in his breast—his pulse at 96—his belly costive—he has very bad nights from the cough—he does not complain of heat or chilliness. I prescribed pulv. fol. digital. in pills with extract. glycyrrhizæ: he was to take a grain and a half twice in 24 hours. I also directed equal parts of of camphorated tincture of opium and oxymel of squills, to be taken at bed time, and occasionally in barley water.

June 20th, I had seen this patient at least four times a week, as he was diligent in his calls at my house since May 21st. I have not yet observed his pulse to be retarded; but yesterday the stricture over his breast was very troublesome—he had shiverings in the day and sweats in the night—his cough as troublesome as ever—he has some sickness at his stomach, with soreness in his chest and between his shoulders—his tongue is white—his belly costive—pulse 120. As this case was favourable, I had expected a different set of symptoms after so long an exhibition of fox-glove; I therefore reflected on the propriety of abandoning it for a more certain practice; but finally resolved to pursue the trial till his stomach was more affected, or his pulse became slower. I directed a blister to be applied to the breast, and the pills to be continued. June 23d, his pulse is slower than it has been, in all probability, since October last—it is only 68. His cough is less frequent in the day, but very troublesome in the night, with perspiration—June 24, pulse 62. I now changed the pills for a tincture of fox-glove, that he might modify the dose according to his pulse, or feelings of the stomach.—June 25, p. 60.—June 26, p. 54. The complaints of his chest and cough are nearly gone; his spitting was yesterday very slightly tinged with blood. Notwithstanding the progress of the case, he has no sickness of the stomach, nor any particular want of appetite. June 29, p. at 42; and with this altered state of the circulation, he has no particular inconvenience to complain of, he only feels generally low. July 7th, his pulse is at 40. I have seen him every day of this month, and have not found it to be more than 50 in any observation. He now took a better diet, with meat and porter sparingly; he continued the drops for several weeks, and his complaints have not returned.

In considering the above cases, a few reflections suggest themselves:

1. The cases of William Harrison and George Underhill were incipient, and the case of Mr. Ward was incipient. The two former were preceded by hæmoptysis, which continued occasionally in the progress: the latter was in my opinion tubercular, and the hæmoptoe was inconsiderable.

However well recommended digitalis may have been in hæmoptoe, it had no material influence in checking the advance of phthisis in Harrison and Underhill, and the symptoms of pulmonic inflammation appeared appeared to increafe under its ufe in their cafes, though the hæmorrhage abated. In the cafe of Mr. Ward, the quicknefs of the pulfe was for many months the ftrongeft indication of this infidious difeafe: we might have fufpected the growing mifchief from his fhort cough, and occafional flight pains of the thorax, accompanied by a pulfe of 100 in a minute during the whole winter, even if his firft attack in September had not been pneumonia, and required repeated bleeding.—A violent cough, with pain and expectoration, and a pulfe of 80 would have given little alarm; when a fhort cough, trifling ftiches, and no expectoration, were very alarming with a pulfe generally above 100, and the heat of the body natural. In this ftate the power of digitalis may be faid to have demanded the confidence of the practitioner in fimilar cafes: the circulation was retarded without inconvenience to the patient, beyond my expectation; in fact, it acted as a regulator of the pulfations of the heart. If Mr. Ward took 30 drops of the tincture twice a day, his pulfe was fure to be nearer 40 than 50 in a minute; if he omitted one dofe in 24 hours, or diminifhed each of the dofes to half, his pulfe increafed to 50 beats or a few more, and during this ftate of the circulation all his complaints difappeared.

The remaining fix cafes were too far advanced to have been capable of cure from any medicine; but it perhaps may be worthy of the humane practitioner's attention, that confiderable diftrefs was added by the effects of the digitalis on the nervous fyftem, and function of the ftomach: fuch effects are indeed looked for in fome inftances, when the pulfe begins to correfpond with the intention of the phyfician. In the cafe of Mrs. B. it is probable, that the continuance of the plan might have brought the pulfe to 60; but fhe was not better for the alteration which had taken place; and the increafe of ftrength after the medicine was left off, too plainly declared the caufe of her rapid decline during its ufe. It was Dr. Withering's opinion, the 23d of May, that the anorexia was not owing to the influence of digitalis, becaufe the pulfe fhould have been flower than its natural ftandard, to correfpond with the ftate of the ftomach as proceeding from that caufe. Facts are however oppofite to this decifion in all the above cafes, excepting in that of Mr. Ward.

But when the function of the ftomach is greatly injured, and little progrefs is made in retarding the actions of the heart, a confiderate man will weigh with great caution the benefit he fpeculates upon, againft the pofitive mifchief in his view. In my opinion the future profpect may may frequently turn the fcale, and the patient may enjoy comparative eafe in his laft days, if he have his digeftion not worfe than his difeafe may make it, and his ftrength and fpirits only wafted in the gentle gradation which is the confequence of phthifis.

In fupport of this cautionary remark, the fecond, third, feventh and eighth cafes are ftrictly in point. I would, however, more particularly attend to the fecond.—Mrs. C. had the appearance of living months longer, if the fullnefs of her mufcles be confidered. An internal hæmorrhage may have cut fhort the difeafe, but there was no evidence of fuch a caufe; and the lofs of irritability feemed to accompany the exhibition of the medicine, with the fame characteriftic marks of its injurious influence on the digeftive organs as appeared in the other patients.

However painful it may be to take this retrofpect, in which the good to be obtained from the remedy does not appear in fo ftriking a light as in former accounts of practitioners, I join in the general acknowledgment of thanks to the ingenious phyficians who have lately excited the attention of their brethren to a remedy for phthifis. It muft however be allowed, that hafty conclufions are of no trifling moment to the peace of relatives and the comfort of patients; and with a view to prevent thefe, I have not been folicitous to conceal my want of fuccefs in the ufe of digitalis.

BIRMINGHAM, *Oct.* 12, 1799.

